# Reproductive Outcomes in Young Breast Cancer Survivors Treated (15–39) in Ontario, Canada

**DOI:** 10.3390/curroncol29110677

**Published:** 2022-11-12

**Authors:** Moira Rushton, Jessica Pudwell, Xuejiao Wei, Madeleine Powell, Harriet Richardson, Maria P. Velez

**Affiliations:** 1 Division of Medical Oncology, The Ottawa Hospital Cancer Centre, Ottawa, ON K1H 8L6, Canada; 2 Department of Obstetrics and Gynecology, Queen’s University, Kingston, ON K7L 3N6, Canada; 3 ICES, Queen’s University, Kingston, ON K7L 3N6, Canada; 4 Department of Public Health Sciences, Queen’s University, Kingston, ON K7L 3N6, Canada; 5 Division of Canadian Cancer Trials Group, Queen’s Cancer Research Institute, Kingston, ON K7L 3N6, Canada

**Keywords:** breast cancer, infertility, early menopause, POI, AYA

## Abstract

We conducted a population-based, retrospective, matched-cohort study to examine the impact of breast cancer diagnosis and treatment on fertility outcomes. Relative risks of infertility, childbirth, premature ovarian insufficiency (POI; age < 40) and early menopause (age < 45) were calculated using modified Poisson regression. Our primary cohort included young women (15–39) with early stage BC diagnosed 1995–2014. Five cancer-free patients were matched to each BC patient by birth year and census subdivision. The BC cohort was further divided by treatment with chemotherapy vs. no chemotherapy treatment. 3903 BC patients and 19,515 cancer-free women. BC patients treated with chemotherapy were at increased risk of infertility (RR 1.81; 95% CI 1.60–2.04), and POI (RR 6.25; 95% CI 5.15–7.58) and decreased childbirth (RR 0.85; 95% CI 0.75–0.96), compared to women without cancer. BC patients who did not receive chemotherapy were also at increased risk of infertility (RR 1.80 95% CI 1.48–2.18) and POI (RR 2.12 95% CI 1.37–3.28). All young BC survivors face an increased risk of diagnosed infertility and POI relative to women without cancer, independent of chemotherapy. These results emphasize the importance of pre-treatment fertility counselling for young women diagnosed with BC.

## 1. Introduction

Breast cancer (BC) is the most common malignancy affecting women under age 40 accounting for ~5% of all breast cancer cases [1,2]. Adolescents and Young Adults (AYAs) aged 15–39 years at the time of cancer diagnosis are a unique population in terms of both the biology of their cancers and the way they experience their cancer journey [3]. Young women with breast cancer have unique concerns regarding fertility, pregnancy and contraception and report having difficulty obtaining information in this regard [3,4]. During the course of a curative-intent treatment plan, early stage (I–III) breast cancer patients will receive a combination of surgery, radiation and systemic therapy. Standard adjuvant treatment is multi-modal and can include hormonal therapy, chemotherapy, and targeted anti-HER2 therapy [5,6,7]. In addition to the systemic therapy options offered to all breast cancer patients, AYA patients are often offered ovarian suppression (surgical or pharmacological) for management of ER positive breast cancer due to increased long-term survival achieved with this strategy [7,8]. 

AYA breast cancer survivors have an increased risk of subsequent infertility diagnosis and/or premature ovarian insufficiency (POI). We have previously reported that compared with non-cancer AYAs, breast cancer survivors have increased risk of infertility diagnosis (RR 1.46; 95% CI 1.30–1.65) and POI (RR 4.37; 3.88–4.93) [9,10]. Several mechanisms may play a role. Chemotherapy is one potential explanation, due to its toxic effect on ovarian follicles leading to POI [11,12,13,14] and has been shown to cause POI in 20–80% of AYA women depending on their age, the use of other treatment in addition to chemotherapy, and physician follow-up [15]. Additionally, women with estrogen sensitive cancers or carriers of BRCA1/2 gene mutations may be advised to have bilateral oophorectomy for ovarian suppression or ovarian cancer prevention, respectively, [4,8,16]. 

The issue of pregnancy after breast cancer is a growing concern. Women are delaying childbearing and family planning such that a greater proportion of AYA breast cancer survivors will be nulliparous. The median age at first pregnancy is steadily rising in Western nations and reached 27 in the United States in 2019 [17]. In clinical practise, patients want to clearly understand their risks of infertility, pregnancy and POI after breast cancer treatment. While we have already studied the risk of POI and diagnosed infertility in AYA cancer survivors, the impact of specific treatment modalities is less well characterized. We hypothesized that breast cancer treatment will increase risk of infertility and POI in young women. The objective of this study was to examine the impact of breast cancer diagnosis and treatment on reproductive outcomes in young breast cancer survivors compared with a matched non-cancer cohort in Ontario, Canada.

## 2. Materials and Methods

### 2.1. Study Design 

We conducted a population-based, retrospective, matched-cohort study on breast cancer patients in Ontario, Canada diagnosed between 1 January 1995–31 December 2014. Ethics approval was obtained from Queen’s University Health Sciences Research Ethics Board, Kingston, Ontario (OBGY-296-16 #6019934, Initial clearance on 12DEC2016).

### 2.2. Data Sources

The cohorts were identified using health administrative databases in Ontario, Canada that contain patient-level information on cancer diagnosis, cancer drug administration as well as inpatient and outpatient data, cancer registry data, and demographics. De-identified databases were accessed on June 11, 2020 through ICES (www.ices.on.ca) and all data sources were linked through a unique encrypted identifier and analyzed at ICES. ICES is an independent, non-profit research institute funded by an annual grant from the Ontario Ministry of Health and Long-Term Care. As a prescribed entity under Ontario’s privacy legislation, ICES is authorized to collect and use health care data for the purposes of health system analysis, evaluation and decision support. Secure access to these data is governed by policies and procedures that are approved by the Information and Privacy Commissioner of Ontario. 

Datasets used to construct the matched cohorts in this study included: Discharge Abstract Database (DAD); National Ambulatory Care Reporting System (NACRS); OHIP database; Same-Day Surgery; Registered Persons Database(RPD); Immigration Refugees and Citizenship Canada Permanent Resident (IRCC-PR) database; ICES derived cohort MOMBABY; ICES Physician Database; Postal Code Conversion File; Cancer Care Ontario Activity Level Reporting; Ontario Cancer Registry (OCR); and the New Drug Funding Program. Details on the databases utilized in this study are presented in the supplemental methods found in Supplementary Material. 

### 2.3. Cohort Creation

**Figure 1:**
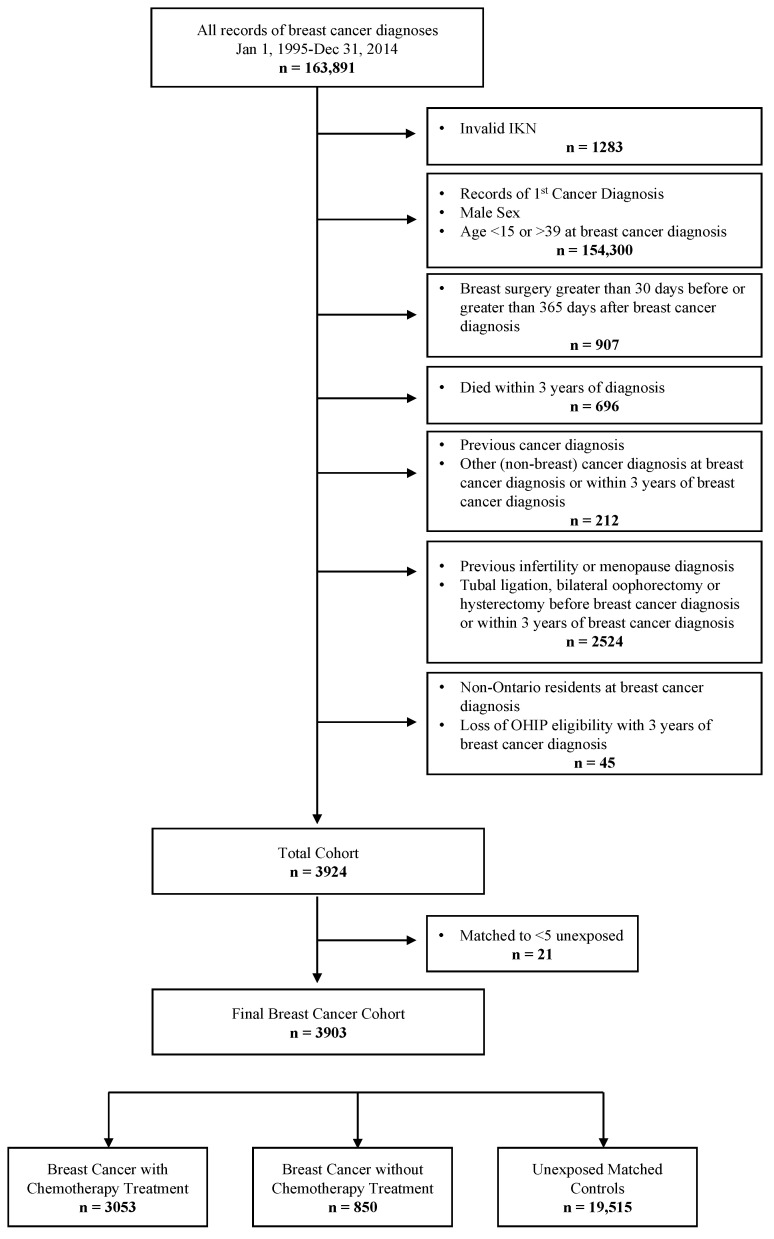
Flow chart demonstrating inclusion and exclusion ceiteria foe cohort design. IKN = ICES Key Number.

Women aged 15–39 who were diagnosed with early stage (I–III) breast cancer between January 1, 1995 and December 31, 2014 were included. The index date for analysis was date of breast cancer surgery (e.g., lumpectomy, mastectomy). This index date was selected to ensure all captured cases were treated with curative intent. Exclusion criteria included: Any prior cancer diagnosis or a second primary cancer diagnosis within 12 months of the index date, women who died within 3 years of diagnosis/index date, stage IV breast cancer at time of diagnosis, prior diagnosis of infertility or menopause, history of prior tubal ligation, bilateral oophorectomy and/or hysterectomy or those same procedures up to 36 months after index date, and missing geographical census data. BC patients who did not have 5 matched controls were also excluded. We further categorized the BC cohort into those treated with or without intravenous chemotherapy. See Figure 1 for cohort inclusion/exclusion flow chart. 

Five cancer-free women from the general population who had no cancer diagnosis prior to the index date were randomly selected without replacement, and were matched on year of birth and census subdivision. Individuals in the cancer-free cohort were assigned an index date based on the date of surgery for their matched case. The cancer-free cohort was subject to the same exclusion criteria as the BC cohort. Individuals who died within 3 years of index date were excluded to remove those with early cancer relapse and/or competing morbidity not otherwise captured in our cohort creation and avoid the bias this may bring to fertility/infertility diagnoses.

### 2.4. Covariates

Age at cancer diagnosis/index, income quintile, rurality index, immigration status, previous pregnancy, and history of endometriosis (ICD-9 617) or polycystic ovarian syndrome (ICD-9 256) were included in the analysis. Data on hormone receptors (estrogen, progesterone), HER2 amplification, and hormonal therapy treatments were not available. 

### 2.5. Endpoints

Individuals in both cohorts were followed from the index date until the occurrence of the primary or secondary outcome or until censored. Censoring occurred at the time of a new primary cancer diagnosis, hysterectomy date, bilateral oophorectomy date, tubal ligation date, loss of Ontario Health Insurance Plan (OHIP) eligibility, death, or maximum follow-up date of 31 December 2019.

Outcomes of interest were: (1) diagnosed infertility defined as the presence of a physician billing code ICD-9 628 in the OHIP database after one year of cancer diagnosis; (2) childbirth defined as delivery of an infant, live or stillborn over 20 weeks gestational age (MOMBABY database); and (3) POI defined as the presence of a physician billing code for menopause (ICD-9 627) before age 40; and early menopause (menopause diagnosis- ICD-9 627–before age 45). 

### 2.6. Statistical Analysis

Descriptive statistics were performed to report baseline characteristics of the cohort. Standardized differences between selected variables were reported for women with and without breast cancer and those differences > 0.10 were considered statistically meaningful in accordance with ICES reporting standards [18]. Modified Poisson regression was used to calculate the relative risk (RR) between exposures and outcomes of interest, adjusted for age at breast cancer surgery, immigrant status, neighbourhood income quintile and prior parity. All RRs are reported with the point estimate along with 95% confidence intervals (CI). The analyses were performed using SAS version 9.4 (Cary, NC, USA) at ICES Queen’s University.

## 3. Results

### 3.1. Patient Characteristics

We identified 3903 women age 15–39 who were diagnosed with early stage breast cancer from 1995–2014 and met our study inclusion criteria for our BC cohort. These were matched to 19,515 cancer-free individuals. Median follow-up time was 12.8 years. Median age for the study population was 36.0 (IQR 33–38) and 78.2% of breast cancer patients received intravenous chemotherapy. Baseline characteristics are described in Table 1. Age was similar distributed as per study design. Women with breast cancer were more likely than women without breast cancer to have given birth before the index date, 50.2% vs. 39.7%. There were less immigrant women in the BC group than Canadian born women. History of PCOS or endometriosis was similar in BC and cancer-free individuals. 

### 3.2. Infertility

**Figure 2:**
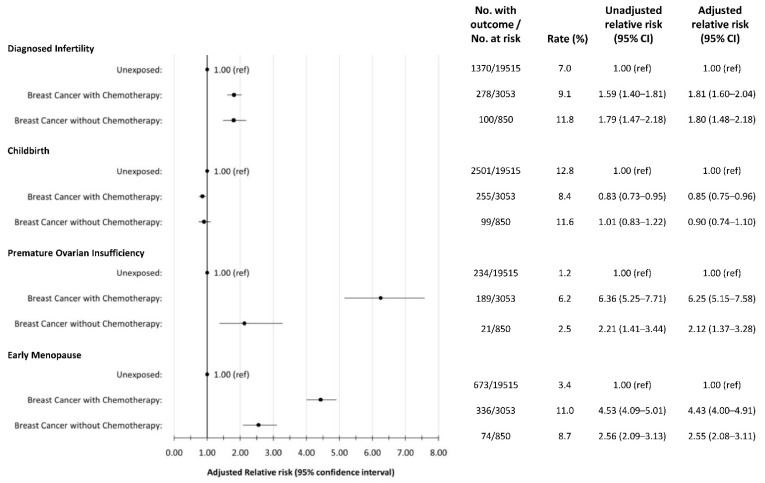
Forest plot depicting rates and relative risks (RR) with 95% confidence intervals for each outcome of interest by exposure and treatment group.

Infertility occurred in 9.1% of breast cancer patients who received chemotherapy, 11.8% of breast cancer patients who did not receive chemotherapy, and in 7.0% of the cancer-free (non-cancer) group. In the Poisson regression model (Figure 2), all cancer patients had an increased RR of infertility diagnosis compared to the non-cancer group with similar adjusted RRs for those treated with chemotherapy (1.81, 95% 1.60–2.04) and those who were not (1.80, 95% CI 1.48–2.18). 

### 3.3. Childbirth

Fewer breast cancer survivors gave birth during follow up than the cancer-free group 9.1% vs. 12.8%. When survivors were categorized into those treated with chemotherapy vs. those were not, 8.4% and 11.6% gave birth during follow-up respectively. In the multivariable model (Figure 2), birth was less likely in the group of breast cancer patients that received chemotherapy, RR 0.85 (95% CI 0.75–0.96).

### 3.4. Premature Ovarian Insufficiency and Early Menopause

POI occurred in 5.4% of breast cancer survivors vs. 1.2% of the cancer-free group. Early menopause occurred in 10.5% of BC patients vs. 3.4% of cancer-free patients. Amongst BC patients treated with chemotherapy, 6.2% experienced POI and 11.0% experienced early menopause compared with 2.5% and 8.4% of those who did not receive chemotherapy. In the multivariable model, the risk of POI or early menopause was significantly increased for all breast cancer patients, regardless of treatment with chemotherapy, compared to the non-cancer group. For those who received chemotherapy the RR of POI and early menopause was 6.25 (95% CI 5.15–7.58) and 4.43 (95% CI 4.00–4.91), respectively. For those who did not receive chemotherapy the RR of POI and early menopause was 2.12 (95% CI 1.37–3.28) and 2.55 (95% CI 2.08–3.11), respectively.

## 4. Discussion

In this study we found that all breast cancer patients are at increased risk of diagnosed infertility, not just those treated with chemotherapy. When compared with an age matched cohort, breast cancer patients treated with chemotherapy have a lower likelihood of giving birth during follow-up compared to women without cancer. BC patients who were not treated with chemotherapy did not have a statistically significant difference in childbirth. The risk of experiencing menopause after a breast cancer diagnosis increases with chemotherapy treatment. Notably, even without chemotherapy treatment there was an increased risk of POI and early menopause among breast cancer survivors. 

Breast cancer is the most common malignancy affecting young women of childbearing age. Unfortunately, in young women, breast cancer is more likely to be high risk and require treatment with neoadjuvant or adjuvant chemotherapy [19] as evidenced in our study where 78% had chemotherapy included in their treatment plan. During the stress of a new cancer diagnosis, long-term health concerns, including fertility preservation, are often overlooked. A 2014 study from the United States estimated that nearly 50% of young women (age < 45) diagnosed with breast cancer, ~10,000 per year, are at risk of infertility due to breast cancer treatment [20]. Based on our findings this is likely an underestimate as we found an increased risk of infertility in all breast cancer survivors, not just those treated with chemotherapy. Current guidelines from the American Society of Clinical Oncology (ASCO) recommend embryo and/or oocyte cryopreservation or use of gonadotropin (GnRH) analogues during chemotherapy for fertility preservation [21]. Guidelines, however, do not always make it into clinical practice. A recent examination of the same Ontario population found that only 4% of AYA breast cancer patients diagnosed 2000–2017 were referred to a gynecologist for pre-chemotherapy fertility counselling [22]. While these rates improved over time, even the most contemporary results saw only 10.7% of patients referred for pre-chemotherapy counselling. 

Counselling patients on the risk of infertility post breast cancer diagnosis is challenging. Our study provides a population-based estimate of infertility risk, childbirth and premature/early menopause in breast cancer patients compared to women without cancer. A similar population based age-matched cohort study from Norway in 2011 demonstrated much lower rates of pregnancy in breast cancer patients (diagnosed from 1967–2004) than was seen in our population with a HR of 0.35 (95% CI 0.27–0.44) compared to women without cancer [23]. A 2021 meta-analysis of pregnancy and pregnancy outcomes after cancer diagnosis of 39 studies including 112,840 BC patients found a 60 % lower rates of pregnancy in BC survivors compared to the general population (RR 0.40; 95% CI 0.32–0.49, *p* < 0.001) [24]. The differences from these large studies and ours may reflect changes in clinical practise or societal differences in family planning and child-rearing between populations. Fertility rates in general have been declining in Canada steadily since 2008, reaching a record low in 2020 of 1.4 live births per adult female age 15–39 [25]. It also demonstrates the importance of performing these analyses in different populations and time periods to understand the contemporary risks facing breast cancer survivors. 

In terms of POI, to our knowledge this is the first study to assess the risk of POI at the population-based level in AYA with BC and the effect of chemotherapy. While it is accepted that chemotherapy possesses an increased rate of POI in 20–80% of AYA [26], our study supports an increased risk of POI even in patients without chemotherapy, likely related to the use of endocrine therapy. However, other contributing factors for this association need to be investigated such as defects in homologous recombination (e.g., BRCA1, BRCA2, PALB2, ATM, CHEK2) known to increase risk of breast cancer [27]. BRCA1 and 2 mutations are known to increase risk of POI due to impaired DNA repair mechanisms [28]. While accepted as a consequence of treatment, POI carries health risks for bone and cardiovascular health. Understanding these risks is important for both patients and providers for long-term health outcomes of these patients. 

Our study has some important strengths including a large sample size, the inclusion of three reproductive outcomes (infertility diagnosis, childbirth, and POI) and the categorization of BC patients according to chemotherapy treatment (yes/no). This is the largest published study to date focusing on infertility diagnosis in relation to cancer treatment as the primary outcome considering cancer treatments; other large studies have focused on childbirth rates as the primary fertility outcome. 

The main limitations of this study stem from the design and use of population-based data which is coded in administrative databases. There is the potential for information bias in this study, since the classification of infertility and menopause outcomes are likely under-reported, since many patients will not seek care for these concerns and therefore, they will not be captured in the health administrative database. We expect this under-reporting (misclassification) to be non-differential between exposure groups, and thus, the relative risks for these outcomes may be attenuated, such that the real effect estimates for breast cancer and infertility or menopause may be even larger than those reported in this article. Due to limitations of the data we were also unable to access information on several important variables which may have led to unmeasured confounding. Specifically, data on estrogen receptor status, endocrine therapy, and specifics of chemotherapy treatment was unavailable. It will be important to continue research in this field to understand whether or not fertility outcomes are impacted by breast cancer stage, subtype and contemporary treatment approaches.

## 5. Conclusions

This population-based cohort study found a significant association between chemotherapy treatment for AYA breast cancer and increased risk of Infertility and POI. There was also a novel finding that women with breast cancer who did not receive chemotherapy were also at a higher risk of Infertility and POI relative to women without cancer; an important detail to inform the care and counselling of AYA breast cancer patients. Further investigation into other mechanisms that can contribute to POI in women with breast cancer is needed. Oncofertility [29] is an important area which requires more attention to optimize care and education of young women with breast cancer.

## Figures and Tables

**Table 1:** Baseline Characteristics and standardized differences between 3903 women age 15–39 years with breast cancer and 19,515 cancer-free women in Ontario, Canada from 1995–2014.

Variable	Value	Chemo No N = 850	Chemo Yes N = 3053	All BC combined N = 3903	Non-Cancer Group N = 19,515	Std Difference *
Age at surgery	Mean (SD)	35.0 (4.1)	35.0 (3.7)	35.0 (3.8)	35.0 (3.9)	0
	Median (Q1–Q3)	36 (33–38)	36 (33–38)	36 (33–38)	36 (33–38)	0
	Min–Max	15–40	18–40	15–40	15–41	
Age group	15–24 - n (%)	18 (2.1%)	38 (1.2%)	56 (1.4%)	302 (1.5%)	0.01
	25–29 - n (%)	78 (9.2%)	265 (8.7%)	343 (8.8%)	1705 (8.7%)	0
	30–34 - n (%)	195 (22.9%)	829 (27.2%)	1024 (26.2%)	5151 (26.4%)	0
	35–41 - n (%)	559 (65.8%)	1921 (62.9%)	2480 (63.5%)	12,357 (63.3%)	0
Parity	Nulliparous - n (%)	500 (58.8%)	1444 (47.3%)	1944 (49.8%)	11,777 (60.3%)	0.21
	Parous - n (%)	350 (41.2%)	1609 (52.7%)	1959 (50.2%)	7738 (39.7%)	0.21
Neighbourhood income quintile	1 - Lowest quintile - n (%)	147 (17.3%)	520 (17.0%)	667 (17.1%)	3944 (20.2%)	0.08
	2 - n (%)	154 (18.1%)	593 (19.4%)	747 (19.1%)	3780 (19.4%)	0.01
	3 - n (%)	195 (22.9%)	657 (21.5%)	852 (21.8%)	3807 (19.5%)	0.06
	4 - n (%)	173 (20.4%)	652 (21.4%)	825 (21.1%)	4134 (21.2%)	0
	5 - Highest quintile - n (%)	181 (21.3%)	631 (20.7%)	812 (20.8%)	3850 (19.7%)	0.03
Immigrant	No - n (%)	663 (78.0%)	2391 (78.3%)	3054 (78.2%)	14,319 (73.4%)	0.11
	Yes - n (%)	187 (22.0%)	662 (21.7%)	849 (21.8%)	5196 (26.6%)	0.11
Rurality	Rural - n (%)	811 (95.4%)	2903 (95.1%)	3714 (95.2%)	18583 (95.2%)	0
	Urban - n (%)	39 (4.6%)	150 (4.9%)	189 (4.8%)	932 (4.8%)	0
Prior Endometriosis	No - n (%)	835 (98.2%)	2993 (98.0%)	3828 (98.1%)	19,261 (98.7%)	0.05
	Yes - n (%)	15 (1.8%)	60 (2.0%)	75 (1.9%)	254 (1.3%)	0.05
Prior PCOS	No - n (%)	829 (97.5%)	3010 (98.6%)	3839 (98.4%)	19,282 (98.8%)	0.04
	Yes - n (%)	21 (2.5%)	43 (1.4%)	64 (1.6%)	233 (1.2%)	0.04

* Differences are between all breast cancer combined and non-cancer patients. Values greater than 0.10 are considered statistically different.

## Data Availability

The data set from this study is held securely in coded form at ICES. While data-sharing agreements prohibit ICES from making the data set publicly available, access may be granted to those who meet prespecified criteria for confidential access, available at www.ices.on.ca/DAS. The full data set creation plan and underlying analytic code are available from the authors upon request, understanding that the computer programmes may rely upon coding templates or macros.

## Supplementary Materials

The following supporting information can be downloaded at: https://www.mdpi.com/article/10.3390/curroncol29110677/s1, supplemental methods
