# Supplementary material for: Reproductive Outcomes in Young Breast Cancer Survivors Treated (15–39) in Ontario, Canada
Source: Curr Oncol. 2022 Nov 12;29(11):8591–9. doi: 10.3390/curroncol29110677 (PMC9689574; doi:10.3390/curroncol29110677)
Supplement: Supplementary file 1 [file curroncol-29-00677-s001.zip › curroncol-1952075-supplementary.pdf]

Supplementary

SUPPLEMENTAL METHODS

Detailed listing of ICES datasets used for this study:

Health Services & Utilization

Discharge Abstract Database (DAD)

The DAD is compiled by the Canadian Institute for Health Information and contains administrative, clinical (diagnoses and procedures/interventions), demographic, and administrative information for all admissions to acute care hospitals, rehab, chronic, and day surgery institutions in Ontario. At ICES, consecutive DAD records are linked together to form 'episodes of care' among the hospitals to which patients have been transferred after their initial admission.

National Ambulatory Care Reporting System (NACRS)

The NACRS is compiled by the Canadian Institute for Health Information and contains administrative, clinical (diagnoses and procedures), demographic, and administrative information for all patient visits made to hospital- and community-based ambulatory care centres (emergency departments, day surgery units, hemodialysis units, and cancer care clinics). At ICES, NACRS records are linked with other data sources (DAD, OMHRS) to identify transitions to other care settings, such as inpatient acute care or psychiatric care.

Ontario Health Insurance Plan (OHIP)

The OHIP claims database contains information on inpatient and outpatient services provided to Ontario residents eligible for the province's publicly funded health insurance system by fee-for-service health care practitioners (primarily physicians) and "shadow billings" for those paid through non-fee-for-service payment plans. The main data elements include patient and physician identifiers (encrypted), code for service provided, date of service, associated diagnosis, and fee paid.

Same-Day Surgery (SDS)

The SDS is compiled by the Canadian Institute for Health Information and contains administrative, clinical (diagnoses and procedures), demographic, and administrative information for all patient visits made to day surgery institutions in Ontario. The main data elements include patient demographics, clinical data (diagnoses, procedures, physician), administrative data (institution/hospital number etc.), financial data, service-specific data elements for day surgery and emergency.

Population & Demographics

Registered Persons Database (RPDB)

The RPDB provides basic demographic information (age, sex, location of residence, date of birth, and date of death for deceased individuals) for those issued an Ontario health insurance number. The RPDB also indicates the time periods for which an individual was eligible to receive publicly funded health insurance benefits and the best known postal code for each registrant on July 1st of each year.

Immigration, Refugees, and Citizenship Canada's (IRCC) Permanent Resident Database

The Ontario portion of the IRCC Permanent Resident Database includes immigration application records for people who initially applied to land in Ontario since 1985. The dataset contains permanent residents' demographic information such as country of citizenship, level of education, mother tongue, and landing date. New immigrants who are currently residing in Ontario but originally landed in another province are not captured in this dataset.

ICES-Derived Cohorts

MOMBABY

The ICES MOMBABY Database is an ICES-derived cohort that links the DAD inpatient admission records of delivering mothers and their newborns. From 2002 onward, this linkage is performed deterministically using a maternal-newborn chart matching number. Prior to 2002, mothers were linked to their children by matching on the institutions they were admitted, their postal codes, and their admission/discharge dates.

Care Providers

ICES Physician Database (IPDB)

The IPDB provides information about all physicians who have practiced in Ontario and is comprised of data contained in the OHIP Claims History Database, the OHIP Corporate Provider Database (CPDB), and the Ontario Physician Human Resource Data Centre (OPHRDC) Database. The database contains information on demographics (age, gender, year of graduation, school of graduation); specialty (functional and certified); location of practice; and measures of physician activity (billings and workload data).

Coding & Geography

Postal Code Conversion File (PCCF)

The PCCF database will link to postal codes within a given cohort and determine other census geographic identifiers such as, dissemination/enumeration area, census division, longitute/latitude, urban/rural flag and neighbourhood income quintile.

Acquired Cohorts & Registries (CCO)

Activity Level Reporting (ALR)

The ALR database is collected by Cancer Care Ontario and represents the basic set of data elements required to produce the quality, cost and performance indicators for the cancer system. The data elements constitute patient level activity within the cancer system focused on radiation and systemic therapy services and outpatient oncology clinic visits. This data is also a key component of the Ontario Cancer Registry (OCR), which registers every malignant neoplasm diagnosed in Ontario.

Ontario Cancer Registry (OCR)

The OCR is collected by Cancer Care Ontario and contains information on all Ontario residents who have been newly diagnosed with cancer ("incidence") or who have died of cancer ("mortality"). All new cases of cancer are registered, except non-melanoma skin cancer.

New Drug Funding Program (NDFP)

The NDFP database is collected by Cancer Care Ontario and captures the use of new, often expensive, cancer drugs. The program was created in 1995 to ensure that Ontario patients have equal access to high-quality intravenous (IV) cancer drugs. The data includes list of drugs, frequency by drug name, patient and treatment data, including size (height, weight) and dosage.
